# The C2 entity of chitosugars is crucial in molecular selectivity of the *Vibrio campbellii* chitoporin

**DOI:** 10.1016/j.jbc.2021.101350

**Published:** 2021-10-27

**Authors:** Wipa Suginta, Surapoj Sanram, Anuwat Aunkham, Mathias Winterhalter, Albert Schulte

**Affiliations:** 1 School of Biomolecular Science and Engineering (BSE), Vidyasirimedhi Institute of Science and Technology (VISTEC), Rayong, Thailand; 2 Department of Life Sciences and Chemistry, Jacobs University Bremen, Bremen, Germany

**Keywords:** marine bacteria, porins, chitoporins, bacterial chitin utilization, chitinolytic pathway, oligosaccharide uptake, biological nanopore regulation, membrane bilayer, membrane pore, carbohydrate metabolism, BLM, black lipid membrane, CNT, carbon nanotube, EOF, electroosmotic flow

## Abstract

The marine bacterium *Vibrio campbellii* expresses a chitooligosaccharide-specific outer-membrane channel (chitoporin) for the efficient uptake of nutritional chitosugars that are externally produced through enzymic degradation of environmental host shell chitin. However, the principles behind the distinct substrate selectivity of chitoporins are unclear. Here, we employed black lipid membrane (BLM) electrophysiology, which handles the measurement of the flow of ionic current through porins in phospholipid bilayers for the assessment of porin conductivities, to investigate the pH dependency of chitosugar–chitoporin interactions for the bacterium’s natural substrate chitohexaose and its deacetylated form, chitosan hexaose. We show that efficient passage of the N-acetylated chitohexaose through the chitoporin is facilitated by its strong affinity for the pore. In contrast, the deacetylated chitosan hexaose is impermeant; however, protonation of the C2 amino entities of chitosan hexaose allows it to be pulled through the channel in the presence of a transmembrane electric field. We concluded from this the crucial role of C2-substitution as the determining factor for chitoporin entry. A change from N-acetylamino- to amino-substitution effectively abolished the ability of approaching molecules to enter the chitoporin, with deacetylation leading to loss of the distinctive structural features of nanopore opening and pore access of chitosugars. These findings provide further understanding of the multistep pathway of chitin utilization by marine *Vibrio* bacteria and may guide the development of solid-state or genetically engineered biological nanopores for relevant technological applications.

Numerous pore-forming proteins (porins) in the outer membrane of Gram-negative bacteria display high substrate specificity (1, 2, 3), with translocation greatly facilitated by weak interactions between pore-lining amino acid residues and permeating molecules such as maltosugars, sucrose, cyclodextrin, phosphate, pyrophosphate, and nucleosides. One of the early studies recording ion current fluctuations in the presence of substrates reported that for the LamB pore (maltoporin) of *Escherichia coli* isomeric structural changes in the carbohydrate substrates had a substantial effect on the interaction of the molecules with the maltoporin (4). Affinity and passage rates were very high for maltose, which has two glucose units linked by an alpha-1,4 glycosidic bond, but rather low for sucrose (fructose and glucose linked alpha-1,4) and lactose (galactose and glucose linked beta-1,4). This observation of the dependence of permeation through LamB on structural details of the interacting sugar substrate was confirmed in a series of detailed structure/function studies by others (*e.g.*, (5, 6, 7, 8)). Substrate specificity was also verified for the bacterial nanopore CymA, from the outer membrane of Gram-negative bacteria *Klebsiella oxytoca* (9) and Vikraman *et al.* (10) described a chemical “passport control” mechanism at the pore opening that regulated the passage of anionic and cationic linear and cyclic oligosaccharides through CymA, selectively. Another porin with high substrate specificity is the sucrose-permeable ScrY channel (11, 12, 13, 14). In the present study the dependence of the chitosugar trafficking activity of a marine bacterium on the fine details of the structure of approaching chitosugars was observed. The effects were, however, in contrast to the studies on LamB and CymA, induced through a much smaller structural variation within the chitosugar ring than the large conformational difference between, for instance, maltose and sucrose or lactose.

The subject of this study is the chitoporin from *Vibrio campbellii* (*V. campbellii*, type strain ATCC BAA 1116), a marine bacterium that is an opportunistic consumer of chitin in tropical seawater and an infectious threat to large-scale shrimp and crab farming. Note that *V. campbellii* was formerly known as *Vibrio harveyi* type strain ATCC BAA 1116 (15): based on the old taxonomy, *Vh*ChiP became the acronym for the chitoporin of the species (16, 17, 18). To be consistent with the terminology in our previous publications and confirm that the chitoporin in this study is the same as that characterized in previous studies, “*Vh*ChiP” has been retained as abbreviation of the studied chitoporin, even though the microorganism of origin is now, in agreement with literature, referred to as *V. campbellii*. The catabolic cascade of environmental chitin utilization by marine *Vibrio* species has been studied for almost a century and was brought to prominence in 1996 by Roseman *et al.*, in five groundbreaking articles (19, 20, 21, 22, 23, 24). The pathway is initiated by extracellularly inducible chitinases, which degrade natural chitin of host crustaceans or chitin sediments. Our own studies of the *V. campbellii* strain BAA 1116 suggested that the microorganism secretes an endochitinase that cleaves accessible chitin into small chitooligosaccharide fragments, (GlcNAc) to (GlcNAc)_6_, with (GlcNAc)_2_ as the main degradation product (25, 26, 27). Uptake of GlcNAc and (GlcNAc)_2_ into the bacterial periplasm is facilitated by general porins while larger fragments permeate the outer membrane through the chitooligosaccharide-specific chitoporin, *Vh*ChiP (16, 17, 18). In the periplasm these longer chitosugars are enzymically cleaved to dimers or monomers, which then enter the cytosol through transporters in the inner membrane and become available for metabolic energy production.

Previously we identified, cloned, expressed, and purified *Vh*ChiP as a trimeric protein pore assembly and analyzed the pore function with di-, tri-, tetra-, penta-, and hexameric chitosugars (16, 17). Using *Vh*ChiP reconstituted in artificial black lipid membranes (BLMs), we found that all chitosugars could move through the three individual pore units of the trimeric chitoporin, and that chitohexaose did this with exceptionally high pore affinity. The strong interaction of *Vh*ChiP with chitohexaose might simply be an adaptation that optimizes uptake efficiency: “ingestion” of a hexose is obviously beneficial for nutrition as it provides six carbohydrate units instead of the smaller number from shorter chitosugars. Further analysis of the ionic current through *Vh*ChiP in the presence of sugars allowed conclusions on the inward and outward movement of chitohexaose, indicating that this chitoporin has multiple internal binding sites (traps) for moving molecules and uses an orchestrated transfer of trapped molecules as an evolutionary strategy to achieve permeation rates high enough for the bacteria to survive in their seawater habitat (18).

In our first *Vh*ChiP studies, we showed that chitosugar uptake and translocation interaction with *Vh*ChiP were very efficient, channel binding increasing with the chain length of the chitooligosaccharide. Chitohexaose, the longest substrate investigated, showed exceptional affinity for *Vh*ChiP. In contrast, maltose or maltooligosaccharides, which are as not natural nutrients for *V. campbellii*, showed negligible permeation through *Vh*ChiP (16, 17). These experiments demonstrated the selectivity of uptake by *V. campbellii*. To confirm further molecular selectivity in chitosugar uptake through *Vh*ChiP, we next compared the interaction of the chitoporin with the natural nutritional substrate chitohexaose and its closest chemical derivative, synthetic chitosan hexaose, used as a molecular probe at various concentrations, pH values, and transmembrane potentials. The two chitosugars are structurally identical apart from their substitution at C2: while chitohexaose has an N-acetyl-function (C2-NHCOCH_3_ or C2-NHAc), in chitosan hexaose the substituent is, depending on pH, an amino (C2-NH_2_) or, after feasible protonation, an ammonium (C2-NH_3_^+^) group. Chitosan hexaose was used to test *Vh*ChiP permeability and to explore the effect of altering the charge on the glucosyl ring. Marked differences were observed in BLM ionic current recordings for *Vh*ChiP that was exposed at various transmembrane potentials to chitohexaose and neutral or cationic chitosan hexaose. Permeation by chitohexaose was independent of pH and transmembrane potential, while chitosan hexaose entry depended on its charge state, regulated by the ambient pH, and on the polarity of the transmembrane potential. This confirmed the strong effect of the acetylation of the C2 nitrogen of a chitosugar on the ability of the molecule to enter and pass through a *Vh*ChiP pore and, in the case of the natural substrate derivative chitosan hexaose, which is normally impenetrant, the possibility to break through an otherwise virtually impenetrable porin gate on protonation and application of an electrostatic driving force.

The passage of ions and small molecules through protein pores in the outer membrane of bacterial cells is a distinct physiological process that is important not only in the maintenance of a healthy electrolyte balance, the supply of anabolic pathways with energy and reactants and the removal of metabolic waste that would otherwise grow to toxic levels, but also for efficient antimicrobial treatment of human bacterial infections (28, 29, 30). A future technological possibility is translation of *Vh*ChiP’s observed characteristics into synthetic solid-state and/or genetically modified biological nanopore devices with integrated molecular entry regulation, a task that biological protein pores such as *Vh*ChiP, but also LamB, CymA and ScrY have realized through evolutionary adaptation of function and that are now a challenge for bio- and nanotechnologists. Feasible applications were recently mentioned and discussed in a series of topical reviews (*e.g.*, (31, 32, 33, 34, 35, 36, 37, 38, 39, 40, 41, 42, 43, 44)) and original articles (*e.g.*, (45, 46, 47, 48, 49, 50, 51, 52, 53, 54, 55, 56, 57, 58, 59, 60, 61)) and include, for instance, use as nanoscopic components of ionic circuits (45, 46, 47, 48, 49), switches of molecular hydrodynamics and transport (50, 51, 52, 53), tools for molecular analytics (54, 55, 56, 57, 58, 59), and nanosieving and nanovalves (60, 61).

## Results and discussion

The chitoporin studied here, *Vh*ChiP, comes from a marine microorganism from a habitat that lacks common sugars but has an abundance of chitin, available either in the shells of crustaceans or as floating particles in seawater or sediments on the ocean floor. *Vh*ChiP would thus be expected to favor chitin-based carbohydrates, and this specificity was indeed observed in our previous studies. A prominent structural feature of chitosugars is the nitrogen substituent at C2, which is acetylated in chitooligosaccharides and deacetylated in chitosan oligosaccharides. The main goal of this study was to define the role of the chemical identity of the C2 substituent in chitoporin uptake and translocation and to investigate the effect of acetylation/deacetylation at C2 on molecular uptake and passage.

The trimeric chitoporin was reconstituted into planar lipid membranes and a transmembrane potential applied to create an ion current. Addition of acetylated chitohexaose ((GlcNAc)_6_) or deacetylated chitosan hexaose ((GlcNH_2_)_6_) induced short ion current blockages or not, and statistical analysis of the fluctuation allowed conclusions on the mode of translocation. As the comparison of structures in Fig. S1*A* shows, the only difference between these sugars is the nitrogen substituents at C2, which are N-acetamido groups (−NHCOCH_3_) in chitin oligosaccharides and unmodified amino (−NH_2_) groups with a pK_a_ of about 6.1 (62) in fully deacetylated chitosan.

**Figure 1:**
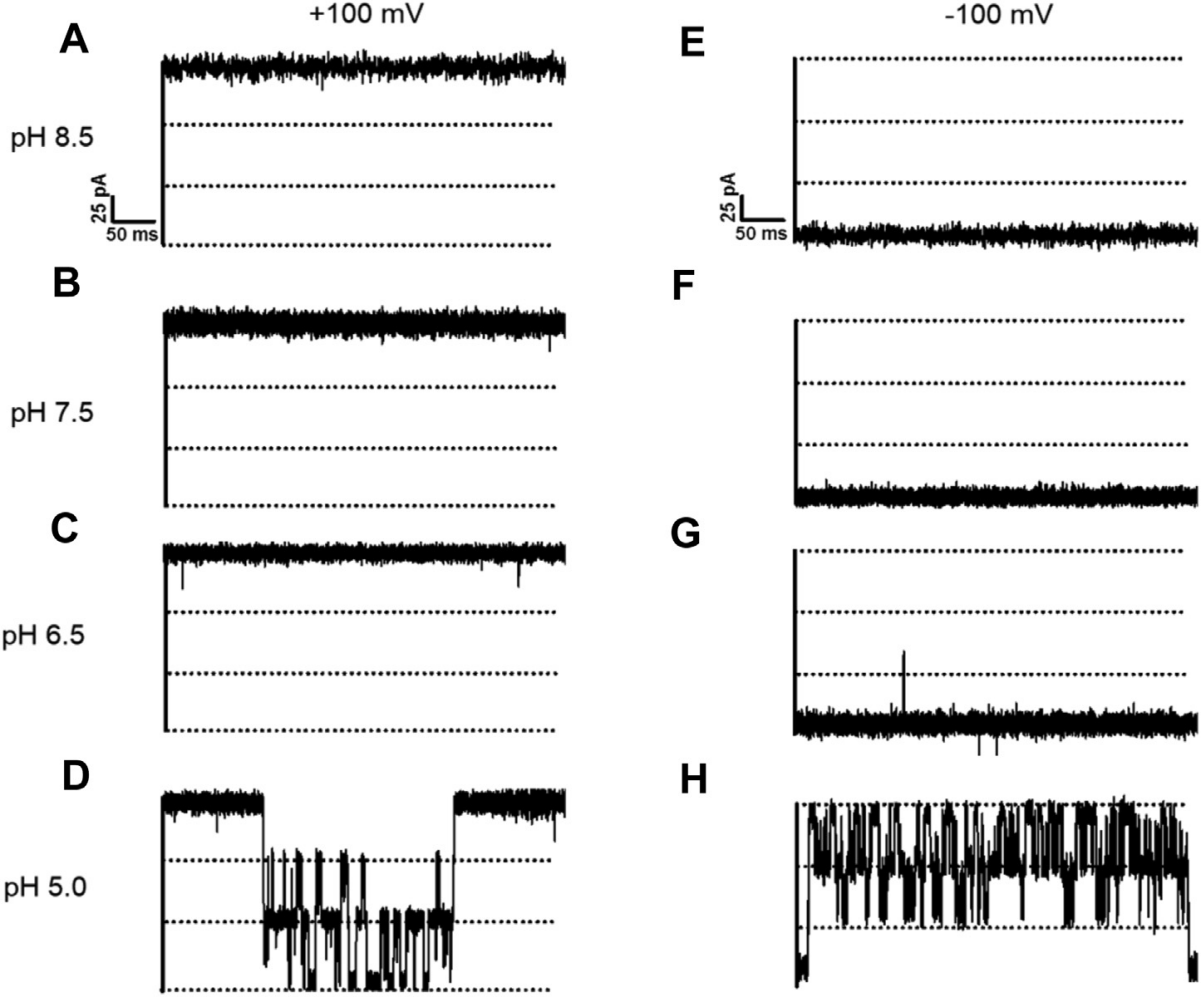
**Effect of pH on chitoporin pore gating in the absence of penetrating chitosugar.** Typical ion current recordings through a single trimeric *Vh*ChiP pore, reconstituted into a lipid bilayer, at various pH values and in the absence of sugar. The chitoporin was inserted from the *cis* side of the membrane and a transmembrane potential of +100 mV (*left panel*) or −100 mV (*right panel*) was applied. The lipid membrane bathing solution contained 1 M KCl with 20 mM HEPES (for pH 8.5 [*A* and *E*], 7.5 [*B* and *F*)], and 6.5 [*C* and *G*]) or 20 mM sodium acetate buffer (for pH 5.0 [*D* and *H*]).

**Figure 2:**
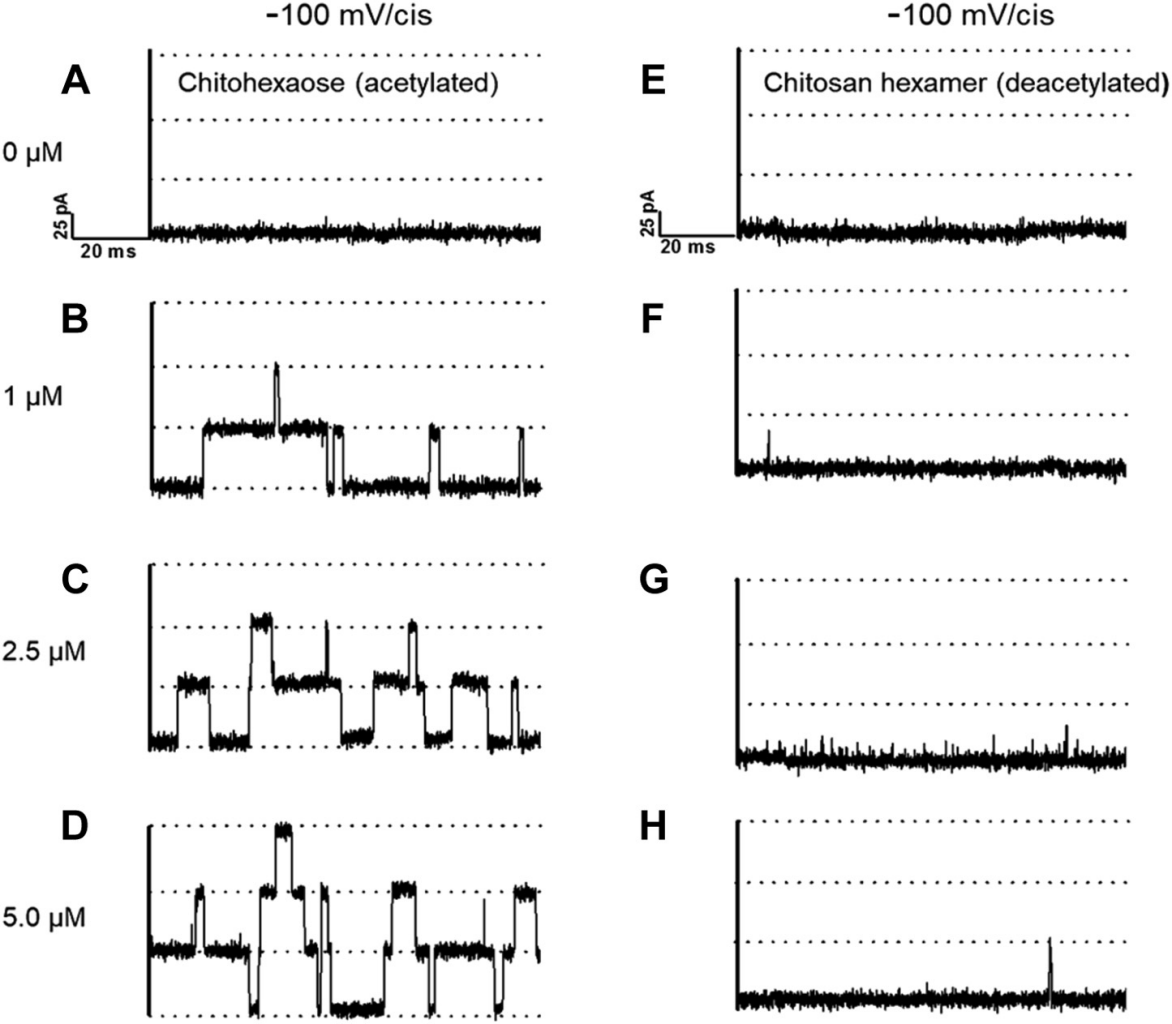
**Effect of N-acetyl functionality at C2 of chitosugars on *Vh*ChiP pore conductance.** Traces are single-pore ion current recordings of single trimeric *Vh*ChiP pores reconstituted into a planar lipid bilayer. The buffer was 20 mM HEPES, pH 8.5 containing 1 M KCl and 0, 1, 5, or 10 μM acetylated chitohexaose (*left panel*, *A*–*D*) or deacetylated chitosan hexamer (*right panel*, *E*–*H*). Chitoporin was added to the *cis* side of the chamber and the transmembrane potential was −100 mV.

We first recorded the open state of *Vh*ChiP in the absence of chitosugar, at electrolyte pH values that would induce the conversion of chitosan hexaose from its largely deprotonated state to the extensively protonated cationic form. At pH 8.5, 7.5, and 6.5 and polarization at −100 and +100 mV, single *Vh*ChiP molecules incorporated into BLMs allowed stable recordings of ion flow (Fig. 1, *A*–*C* and *E*–*G*). Plots of the magnitude of the currents as a function of transmembrane voltage were linear at all three pH values (Fig. S2), and the *Vh*ChiP pore conductance was consistently estimated to be 1.8 nS, in good agreement with our previous results at pH 7.5 (16, 17). Lowering the pH in the *cis* and *trans* BLM chambers to 5.0 at −100 and +100 mV produced a series of spontaneous pore closures and openings (“pore gating”) (Fig. 1, *D* and *H*). The latter observation of low-pH induced *Vh*ChiP gating is believed to be related to a change of the ionization state of protonatable amino acid side chains lining the VhChiP interior and a consequent change in pore shape (63, 64). In earlier studies, *Vh*ChiP pore gating was also verified for instant applications of transmembrane potentials above ±150 mV (16). The addition of chitohexaose at a low concentration (1 μM) on the *cis* side of the BLM produced reproducible uptake-related pore-blocking events, apparent as distinct drops in the transmembrane current (Fig. 2*B*). Increasing the concentration of the chitosugar increased the probability of its molecular uptake and, since *Vh*ChiP is a trimeric pore, simultaneous blockage of two or even all three pore units occurred, full blockage reducing the current to zero (Fig. 2, *C* and *D*).

Addition of the chitosan hexamer to the *cis* side electrolyte under the same conditions did not trigger pore blocking, apart from very rare random events (Fig. 2, *E*–*H*). Uptake and release of chitosan hexaose beyond the time resolution of the BLM measurement were very unlikely because its molecular size is close to that of chitohexaose, and the observed lack of blockage was thus evidence of the inability of neutral chitosan hexaose to enter the pore. Note that as *Vh*ChiP is cation selective (3.2:1) (65, 66) for the indicated negative polarity of the transmembrane potential, the expected electroosmotic flow (EOF) would be a dragging force for molecules approaching and entering the pore. Despite the supporting EOF, no entry of the sugar was detectable. The difference in the interaction of *Vh*ChiP with chitohexaose and chitosan hexaose suggests that the nature of the C2 substitution is a key factor in the ability of an approaching sugar molecule to enter the pore. The presence of N-acetyl groups permits pore entry and defines the high specificity of *Vh*ChiP for acetylated chitooligosaccharides, which are produced by *Vh*ChiP-expressing marine bacteria such as *Vibrio campbelli* and also by *Vibrio cholera* (67), *Serratia marcescens* (68), and *Escherichia coli* (69), through enzymic cleavage of available host chitin. Information on the amino acid sequence alignments between the genes of VhChiP and the other chitoporin is available in the original reports (16, 67, 68, 69).

Chitosan hexaose, on the other hand, with unprotonated –NH_2_ groups, is apparently unrecognizable at the critical C2 site and is therefore unable to enter *Vh*ChiP. This observation accords with our earlier findings from both single-pore and liposome swelling measurements that maltohexaose, with a closely related chemical structure but with hydroxyl substitution of C2, was also inactive in terms of chitoporin entry and membrane current blockage (16).

**Figure 3:**
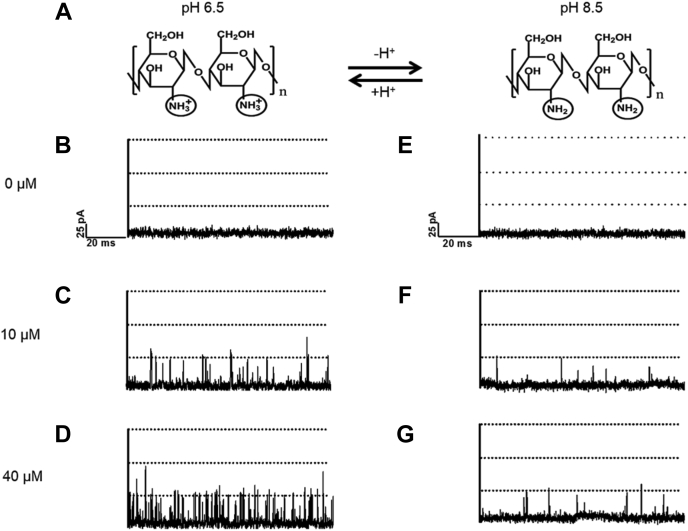
**Effect of pH on translocation of chitosan hexaose through *Vh*ChiP.***A*, Protonation and deprotonation of chitosan hexaose. Traces are ion current recordings from a single trimeric *Vh*ChiP pore reconstituted into a planar lipid bilayer. The electrolyte was 1 M KCl/20 mM HEPES solution, pH 6.5 (*left panel*) or 1 M KCl/20 mM potassium acetate solution, pH 8.5 (*right panel*) without (*B* and *E*) and with 10 μM (*C* and *F*) or 40 μM (*D* and *G*) chitosan hexamer added on the *cis* side. *Vh*ChiP was added to the *cis* side of the chamber and the transmembrane potential for the single pore current recordings was −100 mV.

Entry of chitohexaose into the *Vh*ChiP pore was practically abolished by C2 deacetylation. To inspect the pH dependence of the chitosan hexaose/*Vh*ChiP interaction, BLM trials were performed with 10 and 40 μM chitosugar at pH 8.5 and 6.5. The latter value is just above the pK_a_ of the C2 amino and thus produces significant protonation, relative to the more alkaline condition (Fig. 3*A*). Setting the *trans* electrode at −100 mV produced significant uptake of chitosan hexaose through *Vh*ChiP, visible as transient blockages of the ion current through the BLM-embedded chitoporin (Fig. 3, *B*–*D*). Apparently, the additional coulombic driving force was strong enough to overcome the effect of the missing N-acetyl groups at C2 and admit the charged chitosugar into the pore.

Compared with pore blockage by chitohexaose, signal deflection events with protonated chitosan hexaose were short-lived, suggesting very rapid pore entry and exit by the charged molecule. At both concentrations tested, blocking events were consistently short and rare at pH 8.5 (Fig. 3, *E*–*G*). Deprotonation and elimination of the positive molecular charge removed the electrostatic attraction toward the negative electrode and rendered chitosan hexaose unable to enter the pore, except for some random events.

**Figure 4:**
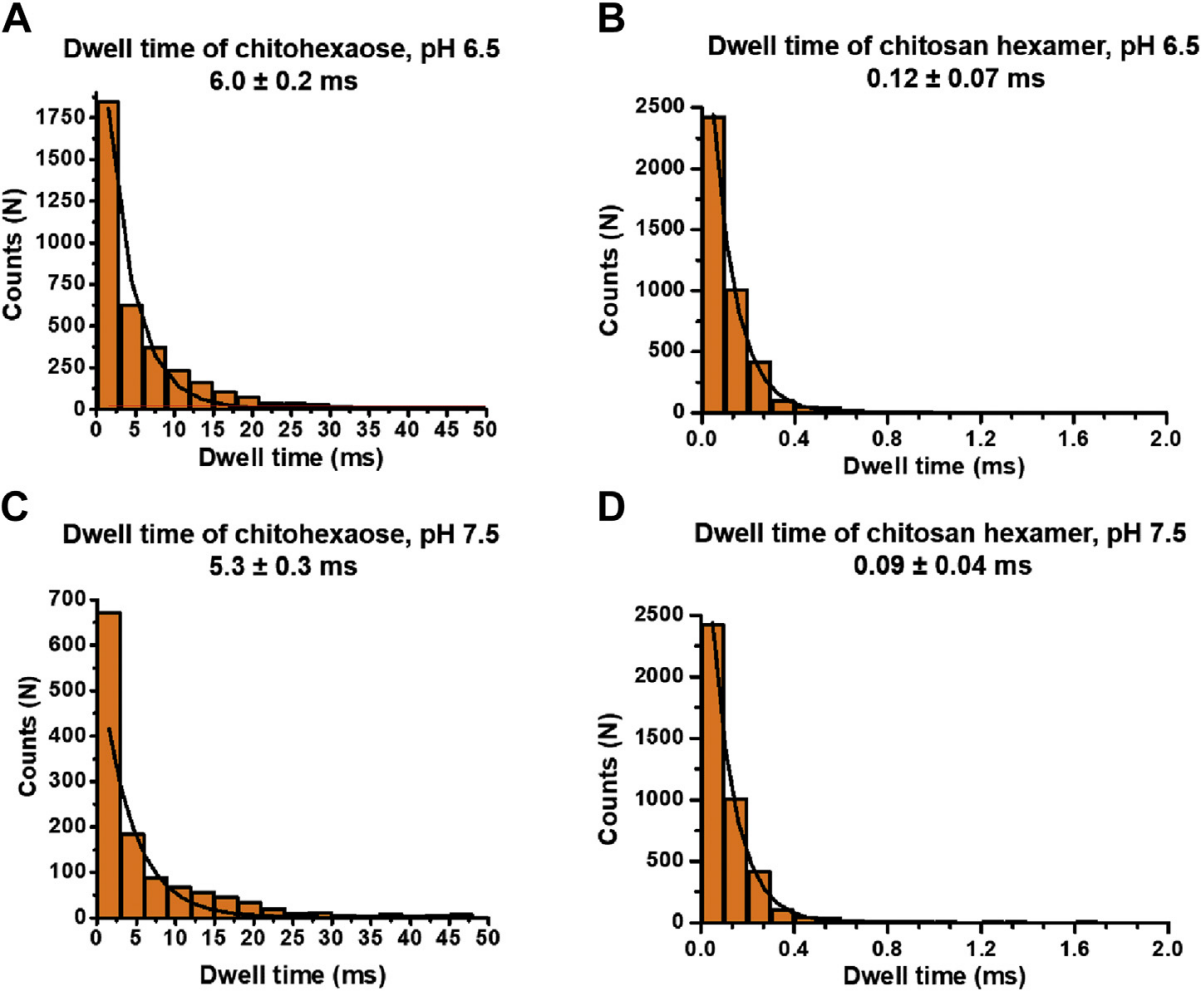
**Dwell time histograms.** BLM recordings were acquired for 120 s. Number of sugar blocking events were obtained by the single pore search algorithm in pClampfit v10.2. The dwell times for both chitosugars were estimated using exponential standard function available in the data analysis software. *A*, chitohexaose pH 6.5, (*B*) Chitosan hexamer, pH 6.5, (*C*) chitohexaose pH 7.5, (*D*) Chitosan hexamer, pH 7.5.

Statistical dwell-time analysis with data from BLM trials with significant (∼30% at pH 6.5) and much less (∼3% at pH 7.5) protonation of the C2 amino groups revealed that the average residence time of chitosan hexaose cations in *Vh*ChiP was 50- to 60-fold shorter than that of chitohexaose (Fig. 4, *A*–*D*). The faster entry and exit of charged chitosan hexaose molecules are expected, as translocation is driven by an electrostatic force and interactions with the amino acids lining the *Vh*ChiP pore wall will be weaker than those with chitohexaose. The much slower movement of chitohexaose through the chitoporin indicates that for this natural *Vh*ChiP substrate molecular affinity to the *Vh*ChiP pore walls decelerates translocation, which is a consequence of the tight binding of the substrate to the pore wall and has presumably evolved to ensure efficient scavenging of chitooligosaccharides from the marine environment.

**Figure 5:**
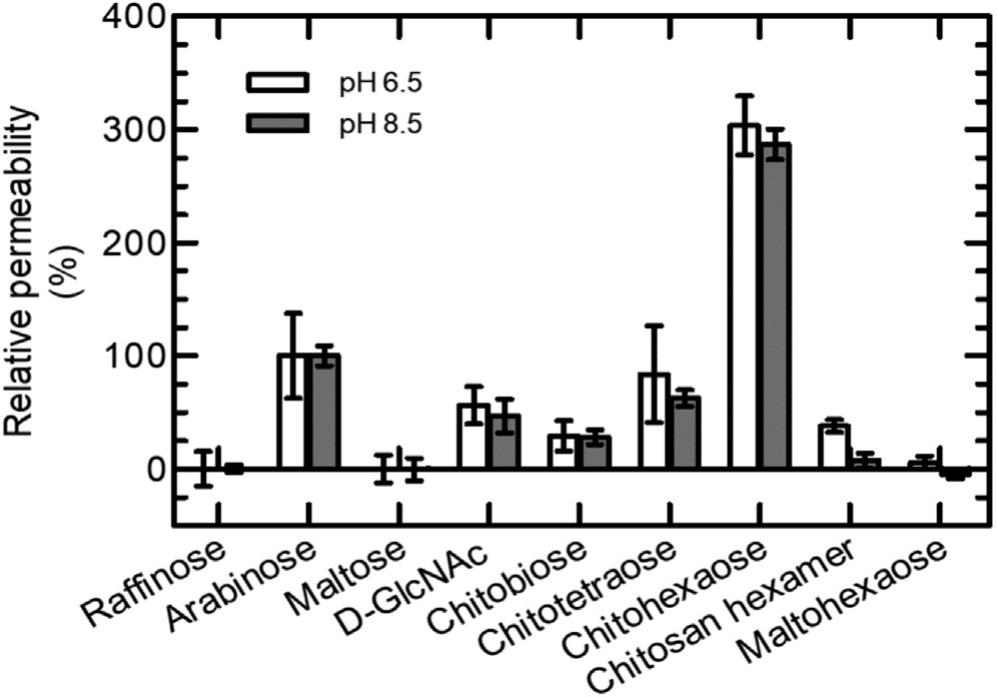
**Bulk permeation of chitosugars and unrelated sugars through *Vh*ChiP into liposomes, at pH 6.5 and 8.5.** Relative permeabilities of the tested sugars at the two pH values were derived from liposome swelling assays. Details of *Vh*ChiP proteoliposome preparation are given in the Experimental procedures section. The permeation rate in an isotonic solution of L-arabinose, the smallest of the sugars tested, was taken as 100%.

The bulk permeation of chito- and other sugars through *Vh*ChiP was tested at low (6.5) and high (8.5) pH values, using a liposome swelling assay. Permeation rates, normalized to that of arabinose, are shown in Figure 5. As expected for the natural *Vh*ChiP substrate, the relative permeation rates of all tested chitosugars were significant at both pH values. Raffinose, maltose, and maltohexaose are not natural chitoporin substrates and showed negligible permeation, and chitosan hexaose permeated less rapidly than the chitosugars. At the same sugar concentration, 5 mM, chitohexaose had the highest relative permeation rate (approx. 300% at pH 6.5 and 280% at pH 8.5). Depending on pH, the shorter chitosugars, D-GlcNAc, chitobiose, chitotriose, and chitotetraose, permeated proteoliposomes through *Vh*ChiP at rates 25 to 80% of that of arabinose. Compared with chitohexaose, the chitosan hexamer permeated poorly at pH 6.5 and 8.5, with rates of about 20% and below 10%, respectively. Overall, the results from liposome swelling assays confirmed those from electrophysiological inspections of *Vh*ChiP–sugar interactions.

The results of titration of *Vh*ChiP with chitohexaose and chitosan hexamer at various concentrations allowed analysis of the binding kinetics of the two sugar species with the *Vh*ChiP pore at pH 6.5 and 7.5 (Table 1). The on- and off-rates for chitohexaose interaction with *Vh*ChiP were independent of the pH and the related binding constants (*K*) were high. Compared with the values for chitohexaose, on-rates for chitosan hexaose were much smaller at both pH values, the off-rates much larger, and the calculated binding constants correspondingly reduced. At pH 6.5, for instance, with the greatest degree of amino-group protonation, chitohexaose showed five times larger on-rate, 50 times smaller off-rate, and a 150-fold greater binding constant. For chitosan hexaose, the on-rate was higher at pH 6.5 than at pH 7.5, while the off-rates were similar, and the relative binding constants were determined by on-rates. Altogether, the titration trials confirmed that the identity of the nitrogen moieties at a chitosugar’s C2 position is crucial for uptake by *Vh*ChiP: −NHCOCH_3_ side chain favors passage into the channel while −NH_2_ side chain reduces entry. Amino-group protonation and imposition of an electromotive force can override this, supporting translocation of the otherwise inactive chitosan hexamer.

**Table 1:** Effect of pH on the binding constant of *Vh*ChiP for uncharged and charged chitooligosaccharides

pH	Chitohexaose			Chitosan hexamer		
	Binding constant (*K*, M^−1^)	On-rate constant (*k*_on,_ 10^6^ M^−1^s^−1^)	Off-rate constant (*k*_off,_ s^−1^)	Binding affinity constant (*K*, M^−1^)	On-rate constant (*k*_on,_ 10^6^ M^−1^s^−1^)	Off-rate constant (*k*_off,_ s^−1^)
6.5	300,000 ± 50,000	51 ± 2.5	170 ± 50	2000 ± 500	11.5 ± 1.6	8300 ± 1400
7.5	350,000 ± 50,000	67 ± 1.0	190 ± 20	300 ± 130	2.9 ± 1.0	9400 ± 800
8.5	300,000 ± 60,000	57 ± 1.8	190 ± 30	-	N.D.^a^	-

BLM data were acquired at −100 mV, with sugar added on *cis* side in buffer containing 1 M KCl. The equilibrium binding constant (K), on-rate constant (k_on_), and off-rate constant (k_off_) were determined from noise analysis, as described in the Experimental procedures section.

a Not detectable.

**Figure 6:**
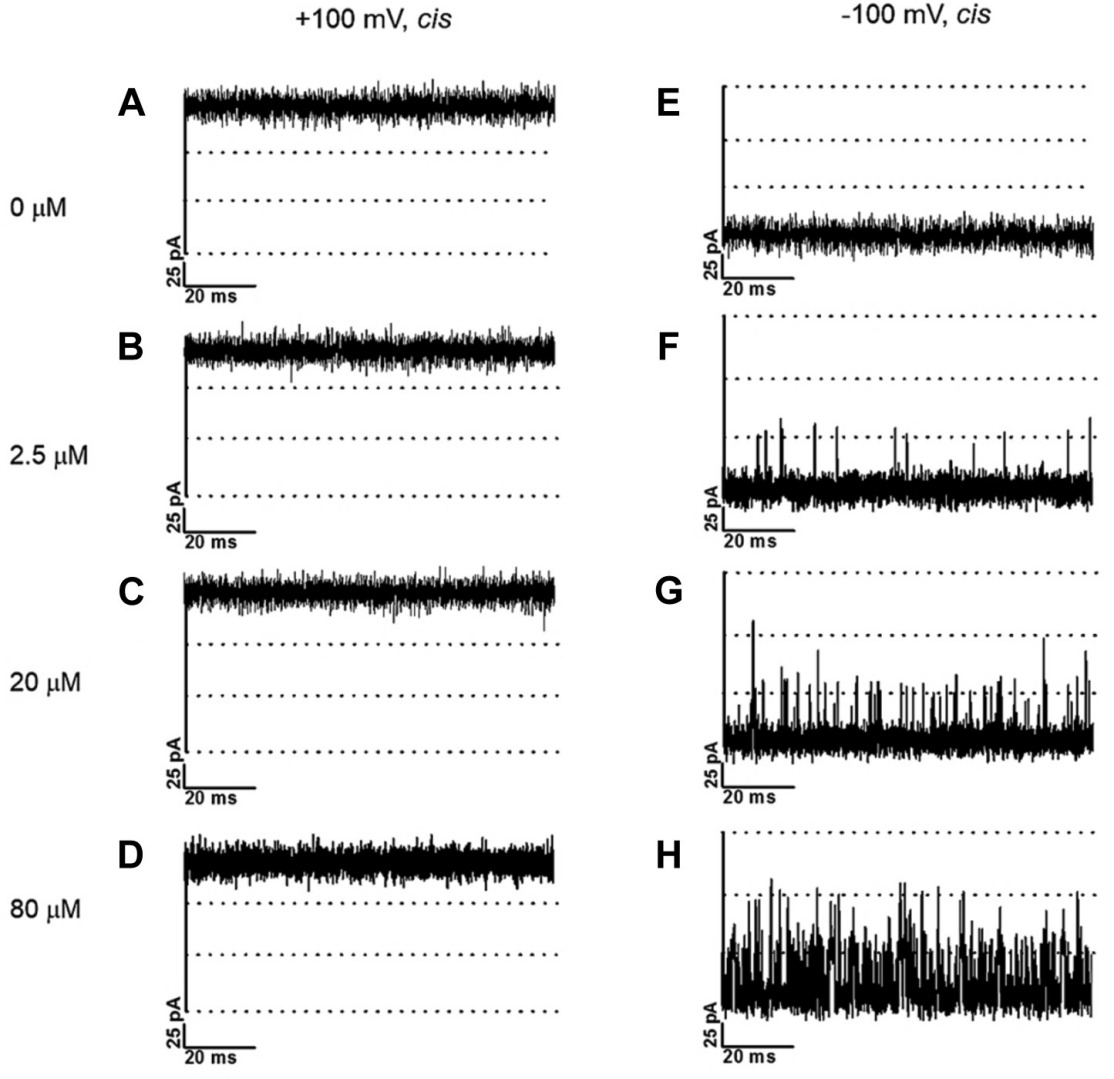
**Influence of polarity of the *trans* electrode on the translocation of cationic chitosan hexamer from the *cis* side of BLMs.** Current recordings from single *Vh*ChiP molecules incorporated in a planar lipid bilayer from the *cis* side and exposed to 20 mM HEPES pH 6.5, 1 M KCl at +100 mV (*A*–*D*), and −100 mV (*B*–*H*). Chitosan hexamer was added at the specified concentrations on the *cis* side of the lipid bilayer.

The BLM measurements presented so far indicate that chitosan hexaose entry into and movement through *Vh*ChiP at significant rates was possible only in the protonated state and with the assistance of coulombic attraction to a negative Ag/AgCl electrode on the outlet side of the protein pore. With protonated chitosan hexaose present in the electrolyte buffer on the *cis* side of the membrane, the electrode polarity on the opposite, *trans* side determined the ability of the cationic sugar to enter and pass through *Vh*ChiP (Fig. 6).

Cationic chitosan hexaose added on the *cis*-side caused no pore blocking at low (2.5 μM), medium (20 μM), or high (80 μM) concentration, when the *trans*-side electrode had the same polarity (+100 mV) and the electrostatic force opposed the movement of cationic sugar from *cis* to *trans* (Fig. 6, left panel). A change of *trans*-side electrode polarity from +100 mV to −100 mV under otherwise equal conditions, however, imposed the necessary coulombic attraction to cationic chitohexaose in the *cis* buffer, enabling frequent inward and outward movements of the sugar and temporary pore occupancies (Fig. 6, right panel). Like the pH change from 8.5 to 6.5, the polarity of the two BLM electrodes can thus work as an on/off switch for pore entry by protonated chitosan hexaose, permeation occurring only with the correct polarity.

**Figure 7:**
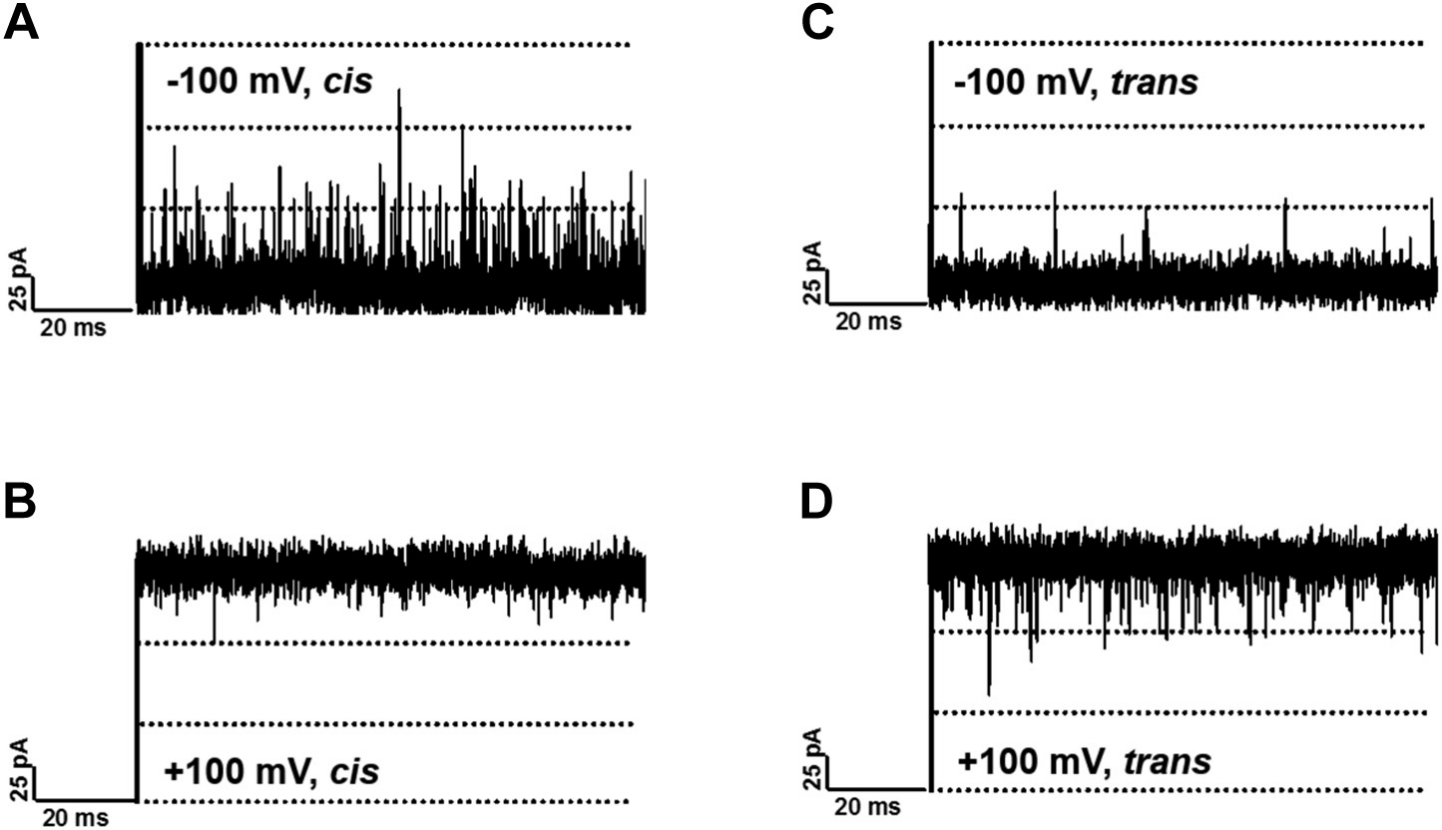
**On/off switching of pore permeability to protonated chitosan hexaose.** A single *V*hChiP pore was incorporated in a planar lipid bilayer and exposed to 1 M KCl, 20 mM HEPES, pH 6.5. 160 μM chitosan hexaose was added on the *cis* (*left panel*) or *trans* (*right panel*) side and a transmembrane potential of +100 mV (*B* and *D*) or −100 mV (*A* and *C*) applied. Statistical trace analysis revealed the number of events per second as: *cis*, −100 mV = 7433 ± 550; *cis*, +100 mV = 650 ± 24; *trans*, −100 mV = 450 ± 30; *trans*, +100 mV = 2250 ± 250.

The efficiency of the electric-field-driven permeation control is further visualized in Figure 7, which shows BLM membrane current recordings in which the side of protonated chitohexaose addition and the polarity of the applied transmembrane potential were varied. Significant pore entry and temporary pore occupation with translocating sugar molecules were observed as transient deflections in the BLM recordings only when the cationic chitosugar and the negative electrode were on opposite sides of the BLM (Fig. 7, *A* and *D*). In contrast, apart from rare events, protonated chitosan hexamer did not move into *Vh*ChiP pores when it was at the same side of the lipid membrane as the negative electrode since the required electrostatic attraction, compensating for the lack of molecular affinity, was absent (Fig. 7, *B* and *C*). This set of experiments does not mimic native conditions, but the results demonstrate the unique possibility to control the flux of molecules and ions through biological nanopores in synthetic membranes through choice of the transmembrane potential and the pH of the electrolyte on either side, if the substrate can, for instance, be adequately positively charged through induced protonation. The application of this principle to modulate nanofluidic ionic diodes, ion-mediated switches, electrostatic nanosieves or ion-mediated nanovalves (31, 32, 33, 34, 35, 36, 37, 38, 39, 40, 41, 42, 43, 44, 45, 46, 47, 48, 49, 50, 51, 52, 53, 54, 55, 56, 57, 58, 59, 60, 61) is feasible but will require cross-disciplinary collaboration. While the potential for such development certainly exists, it is obviously a challenging task to adapt the fundamental properties of the chitoporin reported in this study to final technical applications.

**Figure 8:**
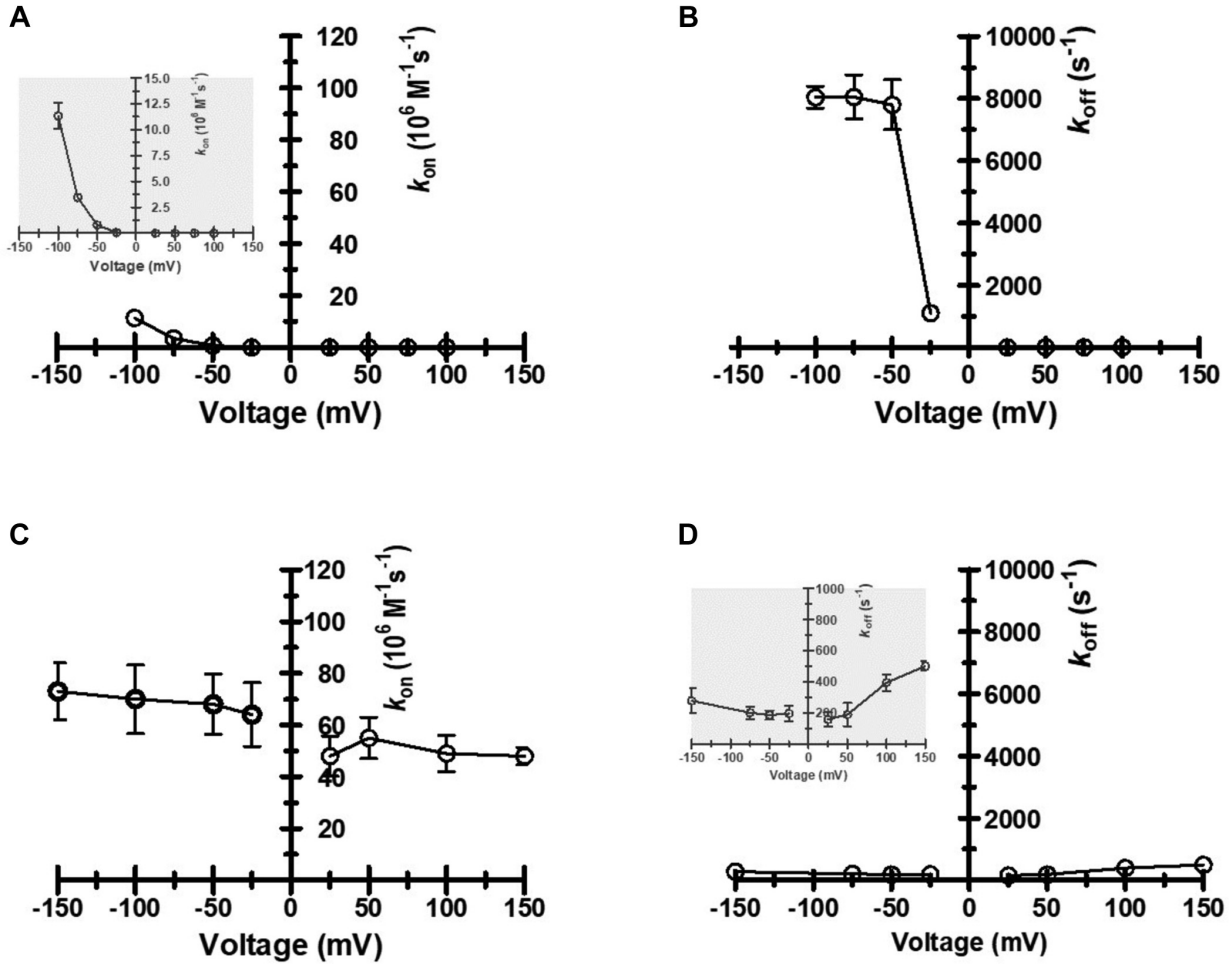
**Potential dependence of the on and off-rates of chitooligosaccharide binding to *Vh*ChiP.** All data were obtained with *Vh*ChiP and the sugars added on the *cis* side of the lipid bilayer, in 1 M KCl, 20 mM potassium acetate buffer (pH 6.5). The data points are means of triplicate measurements at each potential. Plots of (*A*) the on-rate (*k*_on_, 10^6^ × M^−1^s^−1^) for chitosan hexamer, (*B*) the off-rate *k*_off_ (s^−1^) for chitosan hexamer, (*C*) *k*_on_ for chitohexaose, and (*D*) *k*_off_ for chitohexaose against the applied transmembrane potential (V_m_, mV). The kinetic parameters of both sugars were estimated following literature routines (13, 83, 84).

**Figure 9:**
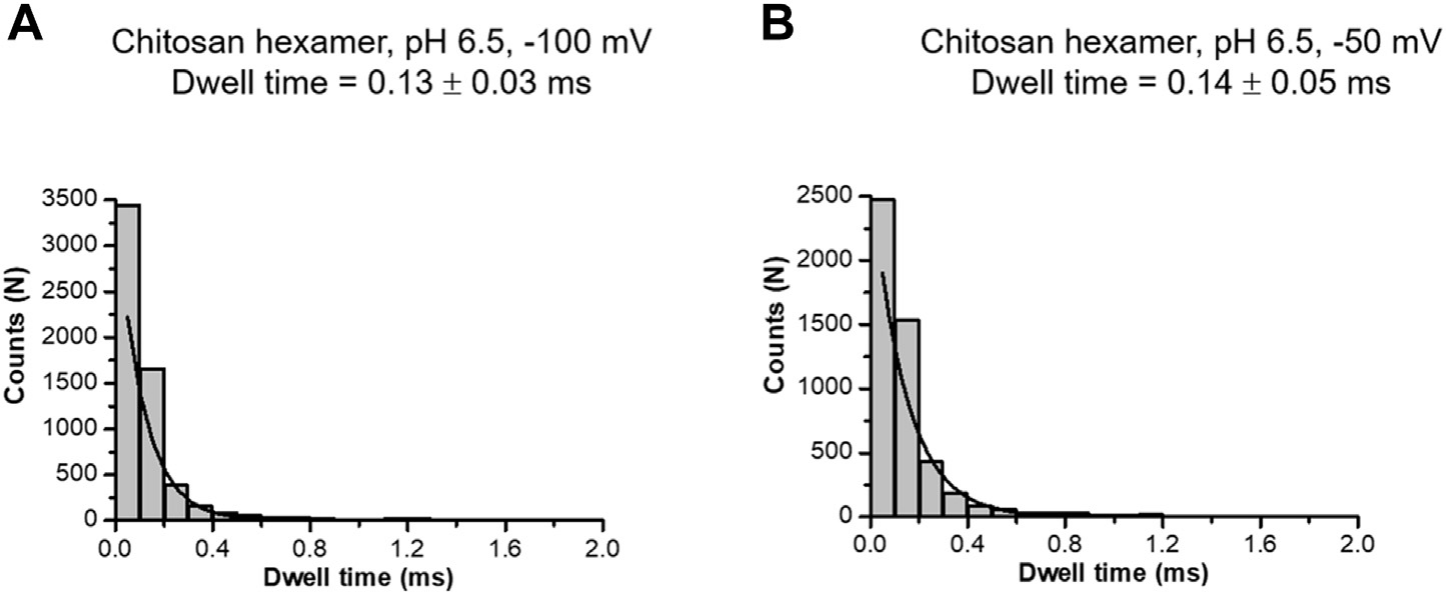
**Effect of external voltages on the dwell time of chitosan hexamer.** The dwell times of the *Vh*ChiP pore at two different voltages were estimated the BLM recordings of 35 μM chitosan hexamer, pH 6.5. Curve fits were performed using exponential standard function available in pClampfit *v*10.2. *A*, −100 mV, *B*, −50 mV.

When cationic chitosan hexaose was added on the *cis* side, on- and off-rates increased as the transmembrane potential was raised from ±25 to ±100 mV (Fig. 8*A*). As expected, cationic chitosan hexamer molecules blocked the pore for short times only at negative *trans* electrode potentials, with a roughly exponential increase in the on-rate with increasing potential (left side of the plot in Fig. 8*A*), while *Vh*ChiP did not interact with the sugar at positive applied *trans* electrode polarization (right side of the plot in Fig. 8*A*). In plots of *k*_off_ *versus* applied transmembrane potential, there was a sudden increase when the potential was changed from −25 to −50 mV, but above that threshold not much further increase (Fig. 8*B*). Obviously, a minimum applied electrical potential is needed to drag charged chitohexose molecules past the molecular control at the *Vh*ChiP gate and through the pore, resulting in translocation. In BLM tests at pH 6.5, with moderately protonated chitosan hexaose, transmembrane potentials of −50 to −100 mV pulled the charged oligosaccharide from *cis* to *trans* side through VhChiP pores, and the variation of the electrostatic force did not affect the dwell time of the molecules (Fig. 9, *A* and *B*). Because of its pronounced affinity for *Vh*ChiP neutral chitohexaose, the natural pore substrate, caused pore current obstructions regardless of the polarity of the transmembrane polarization, and both the on- and the off-rates of chitohexaose were only slightly modulated by the applied potential (Fig. 8, *C* and *D*). *k*_off_ values for chitohexaose were much smaller at negative *trans* BLM electrode potentials than for protonated chitosan hexaose (30-fold lower at −100 mV), indicating much stronger interactions of the translocating molecules with the pore interior and movement that is not driven by electrostatic force. For chitohexaose *k*_on_ was slightly higher at negative potentials (Fig. 8*C*) with the trend reversed for *k*_off_ (Fig. 8*D*, inset). As constant *k*_on_ and *k*_off_ values would be expected for an ideally neutral molecule, these correlations may indicate the influence of partial polarity on the chitohexaose molecule, *e.g.*, around the C2–NHCOCH_3_ groups.

**Figure 10:**
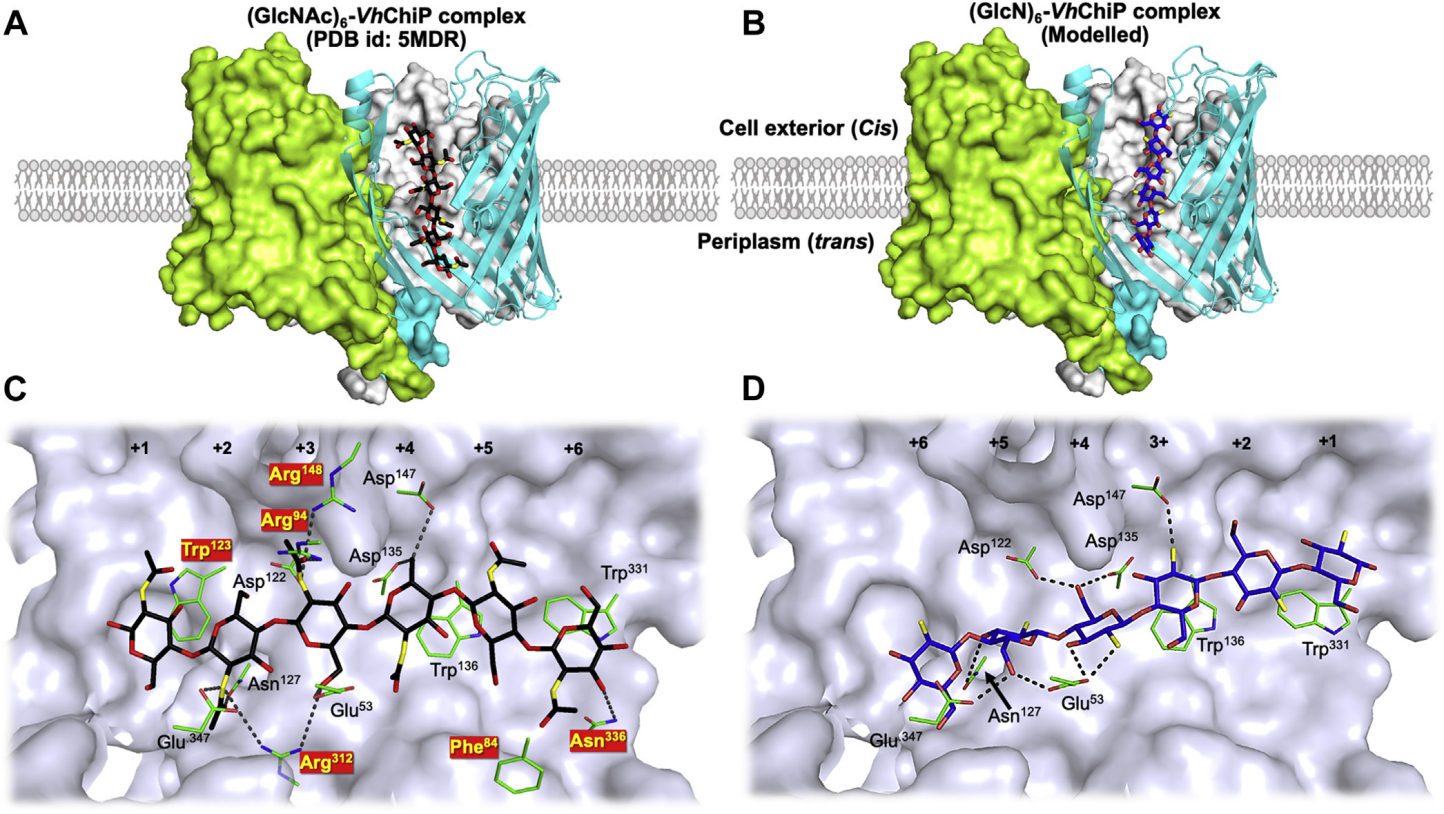
**Structural comparison of*Vh*ChiP complexed with (GlcNAc)_6_ and (GlcN)_6_.** Cartoon representations of the trimeric structure of *Vh*ChiP, displaying the occupation with (*A*) (GlcNAc)_6_ and (*B*) (GlcN)_6_. The molecular interactions between the internal residues of *Vh*ChiP inside the pore and (GlcNAc)_6_ and (GlcN)_6_ are shown in (*C*) and (*D*), respectively. Hydrogen bonds and hydrophobic interactions are set at 3.5 Å, whereas other parameters are set by default. In the chemical structures of the sugars, carbon is presented in *black* for (GlcNAc)_6_ and *blue* for (GlcN)_6_, whereas oxygen and nitrogen are presented in *red* and *yellow*, respectively. Carbon, nitrogen, and oxygen atoms of the surface residues are colored *pale green*, *blue*, and *red*, respectively. *Yellow labels* shown in *red boxes* are identifying the amino acid residues that interact with (GlcNAc)_6_, but not with (GlcN)_6_. The sugar–channel interactions were analyzed by LIGPLOT, and the structures are presented in PyMOL. Numbers +1 to +6 designate the positions of the sugar rings from the nonreducing end to the reducing end.

We previously solved the crystal structure of *Vh*ChiP in complex with its preferred substrate, (GlcNAc)_6_ (Fig. 10*A*) (PDB id: 5MDR, (70)), and to compare the binding feature of deacetylated sugar with the acetylated one, we carried out molecular docking by modeling (GlcN)_6_ inside the *Vh*ChiP pore, as presented in Figure 10*B*. Protein–ligand analysis identified hydrophobic and hydrogen-bond interactions of the chitohexaose molecule with some of the pore-lining residues (Fig. 10*C* and Table S1), which showed that the amino acid residues binding the sugar through hydrophobic interaction are Trp331 (+6 GlcNAc), Phe84 (+6/+5 GlcNAc), Trp136 (+5/+4 GlcNAc), and Trp123 (+1/+2 GlcNAc) and through hydrogen bonding Asn127, Arg312, and Glu347 (+2 GlcNAc), Glu53, Arg94, and Arg148 (+3 GlcNAc), Asp122 (+2/+3 GlcNAc), Asp135 and Asp147 (+4 GlcNAc), and Asn336 (+6 GlcNAc). Within the sugar/porin complex, the C2-acetamido groups of the two central (+2 and +3) GlcNAc sites, in particular, were strongly bonded through molecular interactions. The result of a computational simulation that replaced internal chitohexaose by its neutral chitosan derivative is shown in Figure 10*D*. The change from the native to the artificial ligand resulted in the loss of nine hydrogen bonds for the GlcNAc rings at affinity sites +2, +3, and +6 and a gain of three hydrogen bonds for the GlcNAc rings at sites +4 and +5. In the chitosan hexamer/*Vh*ChiP complex, the hydrogen-bonded association of chitohexaose in positions +2 and +3 is especially destabilized, and the hydrophobic attractions with Phe84 and Trp123 are also missing. The native *Vh*ChiP partner chitohexaose thus has a much stronger association with the protein pore walls than its deacetylated derivative, chitosan hexaose. Obviously, the strong hydrophobic and hydrogen bonding of chitohexaose results in the high value of the binding constant, *K*, as determined by BLM pore current blockade analysis.

*V. campbellii*, the marine bacterium that expresses the *Vh*ChiP chitoporin, lives in saline water. The mean pH of the ocean has fallen from 8.21 in preindustrial times to 8.10 currently and a further drop by 0.3 is likely if the atmospheric CO_2_ concentration rises according to commonly accepted estimates (71). The pKa of fully deacetylated chitosan hexaose is about 6.1 and slightly higher for partially deacetylated chitooligosaccharides. A decrease of ocean pH to values needed for significant chitosan sugar protonation is unlikely, even though human activity has a severe impact on the balance between atmospheric and aquatic CO_2_; in its native habitat *Vh*ChiP would thus encounter only uncharged chitosugars with deprotonated C2-amino groups. The option to pull chitosan hexaose in its protonated state through a *Vh*ChiP pore is scarcely attractive for nanotechnological applications, as here the conditions can be set as required.

The physiological relevance of the proposed *Vh*ChiP entry control is understandable with the information in a publication from Li *et al.* (72), who under the guidance of Saul Roseman identified *cod*, a gene in *V. cholera*e that encodes a chitin oligosaccharide deacetylase (COD), which is expressed when the cells are induced, for instance, by crude crab shells. COD was found to be actively secreted by induced *Vibrio cholerae* at all stages of growth and appeared in the growth medium, but was not cytoplasmic, periplasmic, or inner- or outer-membrane-bound. For chitooligosaccharides (GlcNAc)_n_ (n = 2–6) originating *in vivo* through the action of secreted chitinases on the host’s crustacean matrix, COD catalyzes the deacetylation reaction

$${\left(\text{GlcNAc}\right)}_{\text{n}}\to {\text{GlcNAc}-\text{GlcNH}}_{2}-{\left(\text{GlcNAc}\right)}_{\text{n}-2}+{\text{Ac}}^{-}$$

in the vicinity of the microbes. The mono-deacetylated products of COD activity were claimed to have important roles in cellular communication within a growing bacterial biofilm. If, however, a fraction of chitinase-produced chitooligosaccharides is purposely COD-processed into deacetylated chitosugars for cell communication, rather than cytosolic nutrition, uptake into the periplasm and passage into the cytosol are counterproductive and wasteful. The biological significance of the specific entry control of *Vh*ChiP from a closely related *Vibrio* species is thus likely to reject deacetylated chitosugars and ensure that they are available for cell communication and not consumed by use as a cytosolic energy source.

## Conclusions

**Figure 11:**
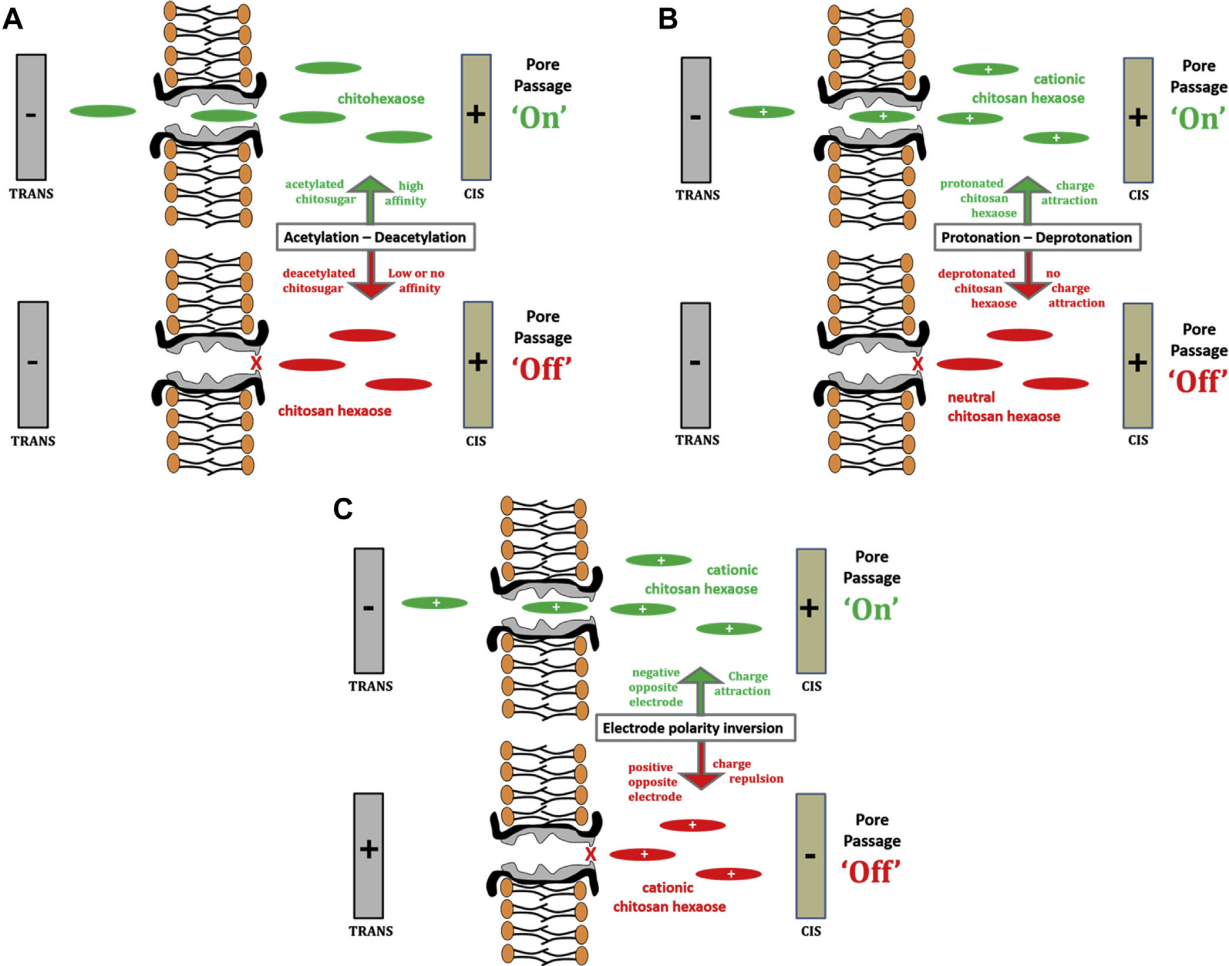
**The impact of acetylation, pH, and transmembrane potential polarity on the translocation of chitohexaose and chitosan hexaose through *Vh*ChiP.***A*, on/off switch determined by the positive or negative “keycard/lock” function of the *C2*-acetamido groups on the chitosugar molecules. *B*, on/off switch of chitosan hexaose pore passage by *cis*/*trans* electrolyte pH. *C*, on/off switch of chitosan hexaose pore passage by electrode polarity.

In its marine habitat, the microorganism acquires nutritional chitosugars through enzymic breakdown of host chitin into short chitooligosaccharide fragments, which are rapidly taken up into the periplasm through *Vh*ChiP in the outer membrane and then into cytoplasm where they serve as an energy supply for metabolic activity. In the present work, the importance of the C2-acetamido group of cleaved host chitin for uptake of derived chitooligosaccharides by *Vh*ChiP was demonstrated. N-acetylated chitohexaose had an exceptionally high affinity for *Vh*ChiP and even at very low concentrations caused frequent transient blockages of single-pore ion currents. In contrast, the deacetylated chitosan hexamer was virtually inactive in terms of pore access and passage. Figure 8 is a schematic summary of the conclusions that can be drawn from our detailed single-pore current analysis of the *Vh*ChiP response to chitohexaose or chitosan hexaose, with pH and transmembrane potentials identified as strongly affecting parameters. N-acetylation/N-deacetylation on C2 was shown to be determinants of VhChiP entry. Possession of the C2–NHCOCH_3_ entity satisfies the molecular “keycard/lock” control in *Vh*ChiP and promotes passage, and replacement of this functionality by C2–NH_2_ results in loss of entry (Fig. 11*A*). The permeation rate of protonated chitosan oligosaccharides with positively charged amino groups was strongly dependent on the polarity of electrodes on either side of the BLM containing integral *Vh*ChiP. Only when the cathode was in the compartment opposite that with the cationic sugar addition did chitohexaose pass through *V*hChiP at significant rates. The driving force for cationic chitosugar entry is electrostatic attraction to the electrode on the side of the pore outlet, rather than substrate structure and a pronounced chemical affinity for the pore (Fig. 11*B*). Reversal of the electrode polarity virtually stopped electrostatically driven movement of cationic chitosan hexaose through the VhChiP pore (Fig. 11*C*).

The use of charged chitosan hexamer as a probe of *Vh*ChiP pore activity and a comparison of the affinities of chitohexaose and chitosan hexaose for amino acid residues in the *Vh*ChiP pore interior revealed an intrinsic functional-group selectivity and charge asymmetry inside the protein pore. The natural substrate for *Vh*ChiP is N-acetylated chitooligosaccharide, while the synthetic chitosan analogue has virtually no porin affinity. Protonation of the amino groups in the chitosan sugar structure allows its passage through chitoporin by cation formation and coulombic attraction, but only with a transmembrane potential of the correct polarity, which regulates pore entry by the cationic chitin oligosaccharide. The ability of a chitoporin to allow or prohibit substrate uptake and passage based on the chemical identity of one group is a striking property of the bacterial outer membrane protein. *Vh*ChiP/chitosugar interaction illustrates the evolution of molecular machinery with extraordinary functional performance and optimization of the cell membrane for bacterial survival. The study findings agree with recent conclusions on electrostatically driven pore permeation through porins in other microorganisms (10, 73, 74, 75). From the viewpoint of the nanotech industry they may be seen as guidance for construction of solid-state or genetically modified biological nanopores for technology and analytical chemistry applications. The exact structural mechanism of the preference for acetylated over deacetylated chitosugar uptake, namely the details of the identity check at the chitoporin gate, has yet to be determined. As *V. campbellii* in its marine environment is not challenged with the uptake of deacetylated chitosugars, the observed phenomenon may not be essential for the survival of the microorganism. However, there is a clear difference in the strength of chitohexaose and chitosan hexaose interaction with *Vh*ChiP, and the uptake and translocation of the chitosan hexaose are strongly pH- and voltage-dependent. The distinct entry regulation of the *Vh*ChiP nanopore could, upon full structural and chemical characterization, be a template similar to the alpha-hemolysin/helicase biomolecule couple (76, 77, 78, 79), which is a combination of two proteins that does not exist in nature but has been incorporated in artificial lipid membranes for the technical task of DNA sequencing. Though there is no explicit role for it in nature and its exact mechanism is not yet known, the regulation of *Vh*ChiP/chitosan hexaose interaction has potential technical applications. Observations from the plethora of published studies (31, 32, 33, 34, 35, 36, 37, 38, 39, 40, 41, 42, 43, 44, 45, 46, 47, 48, 49, 50, 51, 52, 53, 54, 55, 56, 57, 58, 59, 60, 61) are seen here as possible starting points in explorations of such a nanoscopic functional unit. Recently, carbon nanotubes (CNTs) were successfully incorporated in planar lipid bilayers and shown to function as synthetic porins (80, 81, 82). Attachment of recognition sites, such as those apparently present in *V*hChiP, to CNT tube openings could pave the way to control of their molecular passage behavior and selectivity. Our future work will use a combination of targeted amino acid deletions and/or mutations around the pore opening region with functional BLM- and crystal structure analysis to elucidate the details of the identity check (control) of molecular chitoporin entry.

## Experimental procedures

### *Vh*ChiP electrophysiology

The biochemical synthesis, isolation, and purification of *Vh*ChiP and all BLM measurements for the chitoporin followed previously reported procedures (16). The BLM measuring unit, graphically displayed in Fig. S3, had two equal electrolyte chambers separated by a 25-μm-thick Teflon film with a central aperture of 60 to 100 μm diameter. Before use, the rim of the small hole was pretreated with 1 μl of 1% (v/v) hexadecane in hexane (Sigma Aldrich Corp) to facilitate stable BLM formation on a uniform hydrophobic coating. The two half-cells were then filled with equal volumes of electrolyte solution (1 M KCl in 20 mM HEPES or potassium acetate buffer), adjusted to the required pH (5.0, 6.5, 7.5, or 8.5), and equipped with Ag/AgCl electrodes at either membrane side (World Precision Instruments). A virtually solvent-free planar lipid bilayer was finally formed from a 5 mg ml^−1^ solution of diphytanoylphosphatidyl-choline (DPhPC, Avanti Polar Lipids, Inc) in pentane (Sigma Aldrich Corp) by the classical Montal Mueller technique. In typical *Vh*ChiP tests, small volumes of stock solutions of the trimeric protein (50–100 ng ml^−1^) were added to the electrolyte on the *cis* side and transmembrane potential elevations (*e.g.*, +200 mV) were applied to aid the chitoporin insertion into the bilayer membrane. Recognition of a *Vh*ChiP insertion into a BLM membrane was followed by immediate gentle stepwise dilution of the protein-containing *cis*-side electrolyte, to lower the probability of follow-up insertions. For chitosugar translocation studies, chitohexaose or chitosan hexaose (Dextra, Science and Technology Centre) was added at various concentrations to the *cis* or *trans* electrolyte. Single-pore ion flow (current) through inserted *Vh*ChiP was then measured with an Axopatch 200B electrophysiology amplifier (Molecular Devices) with the transmembrane potential clamped against the ground (*cis*) electrode. The picoampere-sized electrode responses were digitized with the system’s AD/DA board (Digidata 1550B, Molecular Devices) and monitored and stored with the amplifier’s own data acquisition software (pClamp 10.6, Molecular Devices). With a low-pass Bessel filter and sampling frequency of 10 and 50 kHz, the pore current recordings lasted 2 min.

Equilibrium chitosugar-binding constants (*K*, M^−1^) were estimated from the reduction of the average ion conductance with increasing substrate levels, using a simple binding expression (13, 83). Through statistical analysis of single chitoporin pore currents and estimates of the average residence (dwell) time (s^−1^) of sugar molecule in the pore, the rates of pore association (*k*_on_, M^−1^ s^−1^) and dissociation (*k*_off_, [s^−1^]) were determined as described by Kullman *et al.* (84). For chitosan hexaose, *k*_on_ was derived from the (number of blocking events per second)/3 × [c], and *k*_off_ from the relationship *k*_off_ = *k*_on_/*K*, since the dwell time of this sugar could not be determined with confidence from the acquired data because of time resolution of the BLM setup.

### Liposome swelling assay

Bulk permeation of different sugars through *Vh*ChiP was tested using a liposome swelling assay (85) to verify the translocation ability of chitinous oligosaccharides. Soybean L-α-phosphatidylchloline (Sigma-Aldrich) (20 mg ml^−1^, freshly prepared in chloroform) was used to form multilamellar liposomes and 15% (w/v) dextran (MW 40000) was entrapped in the liposomes. The homogeneity of obtained liposomes preparations was checked with a Zetasizer Nano System (Malvern Instruments Ltd). The diameters of the dextran-filled liposomes varied from 100 to 1000 nm. The purified *Vh*ChiP (100 ng) was reconstituted into liposomes as described previously (10). The isotonic solute concentration was determined with different concentrations of raffinose solution (prepared in 20 mM HEPES buffer, pH 6.5 or 8.5) added into the proteoliposome suspensions. The sugar substrates (5 mM) were prepared in raffinose solution, to give a total sugar concentration that was isotonic. Twenty microliters of liposome or proteoliposome solution was diluted into 500 μl of the isotonic test solution in a 1-ml cuvette and mixed manually. The initial swelling rate upon addition of the isotonic sugar solutions was monitored using a UV-Vis spectrophotometer with the wavelength set at 500 nm. The absorbance change over the first 60 s was used to estimate the permeation rate (s^−1^) following the equation: Ø = (1/A_i_) dA/dt, in which Ø is the swelling rate, A_i_ the initial absorbance, and dA/dt the rate of absorbance change during the first 60 s. The swelling rate with each sugar was normalized by setting the rate for arabinose (M_r_ 150) to 100%.

### Molecular docking

The crystallographic data for *Vh*ChiP in complex with chitohexaose with 1.90 Å resolution was obtained from the Protein Data Bank (PDB id: 5MDR) (70). Molecular docking was initiated by introducing hydrogen atoms into the *Vh*ChiP molecule using the ionization and tautomeric states inferred by the GOLD program (86), while all water molecules, ions and ligands were removed from the crystal structure. In the docking procedure, other parameters were adjusted by default. The automatic genetic algorithm (GA) parameter setting was used in all the GOLD docking calculations. To define the key sugar binding sites, only amino acid residues of *Vh*ChiP within a 6 Å radius from the center of the bound chitohexaose were considered. Docking trials were carried out by replacing chitohexaose by nonprotonated chitosan hexamer. The chemical structure of chitosan hexamer was then taken from HyperChem professional (87). The scoring function used in all docking calculations was “ChemPLP,” available in GOLD v.5.3. The ligands were visualized, and their geometries were optimized to relaxed forms by the program Discovery Studio Visualizer v.16 (88). Iterated cycles of docking simulations were performed in GOLD, generating 100 possible ligand conformers. All the GOLD docking calculations and were further investigated for protein–ligand interactions. The other parameters of docking runs were set up in accordance with the default values. The conformer with the highest ChemPLP docking score of 97.757 was selected for protein ligand analysis.

## Data availability

All data are contained within this article. Source files are available on request.

## Dedication

In honor of the 100th anniversary of the birth of the late Saul Roseman (March 9, 1921–July 2, 2011), the Ralph S. O'Connor Professor of Biology, Emeritus, at the Johns Hopkins University in Baltimore, Maryland, dedicated deviser of the catabolic cascade of environmental chitin utilization by marine *Vibrio* species.

## Supporting information

This article contains supporting information.

## Conflict of interest

The authors declare that they have no conflicts of interest with the contents of this article.
