# Supplementary material for: The C2 entity of chitosugars is crucial in molecular selectivity of the Vibrio campbellii chitoporin
Source: J Biol Chem. 2021 Oct 27;297(6):101350. doi: 10.1016/j.jbc.2021.101350 (PMC8608610; doi:10.1016/j.jbc.2021.101350)
Supplement: Supplemental Figures S1–S3 and Table S1 [file mmc1.pdf]

Supplementary Information

Molecular selectivity by a marine *Vibrio campbellii* chitoporin: the chitosugar's C2-identity is crucial for passage through the pore

Wipa Suginta[1,*], Surapoj Sanram[1], Anuwat Aunkham[1], Mathias Winterhalter[2], Albert Schulte[1,*]

[1]School of Biomolecular Science and Engineering, Vidyasirimedhi Institute of Science and Technology, Wangchan Valley, Rayong 21210, Thailand

[2]Department of Life Sciences and Chemistry, Jacobs University Bremen, Bremen 28759, Germany

This supporting information file contains the following six items:

1. The chemical structure of chitohexaose and chitosan hexaose (Figure S1)
2. The current-voltage (I–V) curves for *Vh*ChiP in a planar lipid bilayer at various pH (Figure S2)
3. Graphical display of the workstation for BLM measurements (Figure S3)
4. A list of hydrogen bondings and hydrophobic interactions between chitosan hexaose and chitohexaose with aminoc acid residues of the pore interior of wild-type *Vh*ChiP

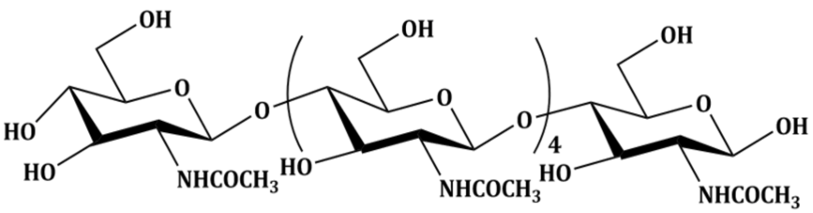

Chitohexaose $(GlcNAc)_6$

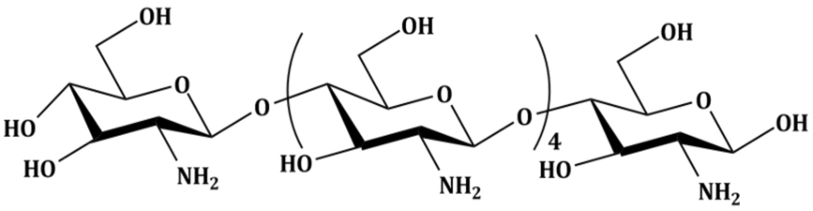

Chitosan hexamer $(GlcN)_6$

Figure S1: Chemical structure of chitohexaose and chitosan hexamer. The two structures were created and displayed using ChemDraw v.9 (Perkin Elmer, Waltham, MA, USA).

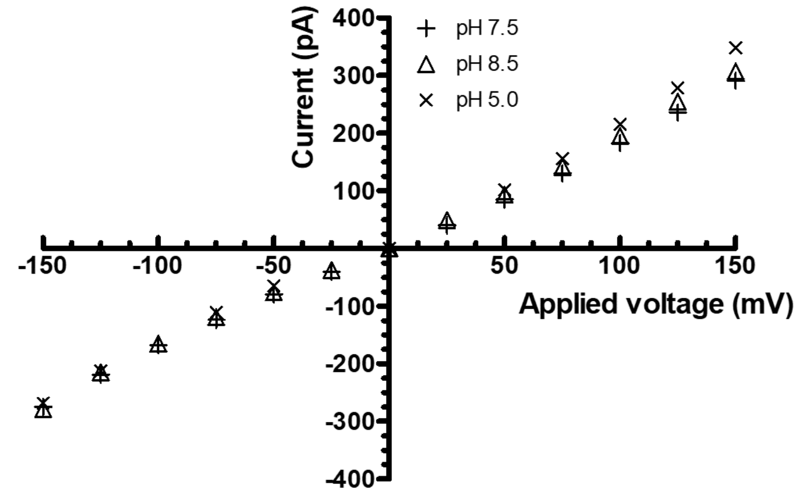

Figure S2: Current-voltage (I–V) curves for VhChiP in a planar lipid bilayer at various pH. *Vh*ChiP was inserted from the cis side of the lipid bilayer. The measuring solution was 1 M KCl, 20 mM HEPES (for pH 7.5 and 8.5) or 20 mM potassium acetate buffer (for pH 5.0). Adjustment of pH used additional volumes of 1 M KOH. Plotted data points are mean values of triplicate measurements at each potential and pH value.

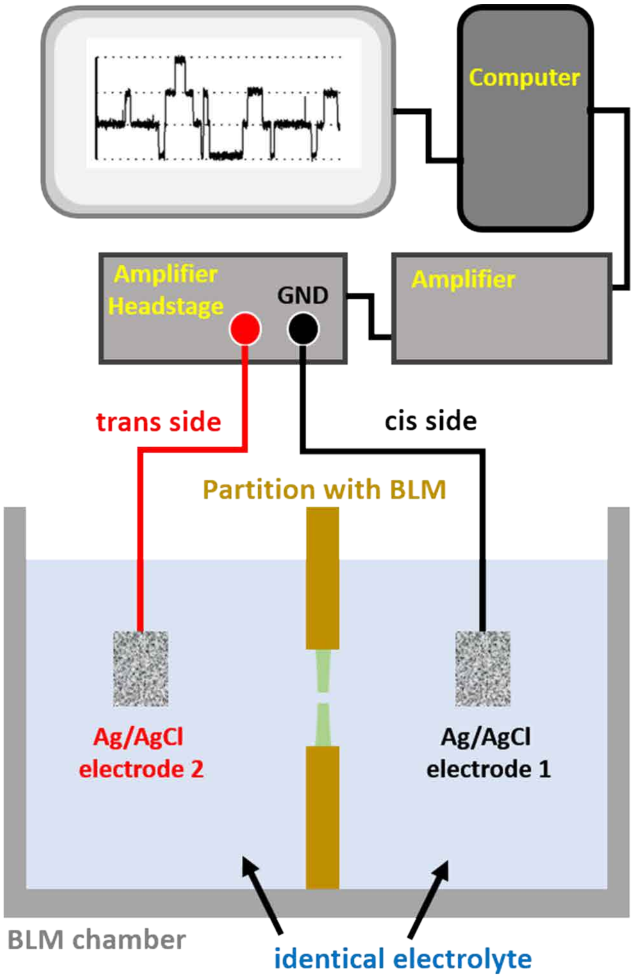

Figure S3: Schematic of the BLM workstation as it was used for the recording of ion flux through *Vh*ChiP chitoporins that were reconstituted into black lipid membranes. The 'cis' side silver/silver chloride electrode was connected to the ground (GND) port of the amplifier headstage while the 'trans' equivalent was at the opposite side of the black lipid bilayer.

Table S1: Hydrogen bonding and hydrophobic interactions details in substrates binding to *Vh*ChiP.

			Docking: *Vh*ChiP wild type and (GlcN)$_6$	*Vh*ChiP WT complex with chitohexaose (5MDR)
			Donor….Acceptor for hydrogen bond,Residues for Hydrophobic interaction (Å)	Donor….Acceptor for hydrogen bond, Residue names for Hydrophobic interaction (Å)
**Affinity sites**	+1	Hydrogen bonding	-	-
		Hydrophobic interaction	Trp^{331}	Trp^{123}
	+2	Hydrogen bonding	-	Asn^{127}….O-acetamido (2.73 Å)., , Arg^{312}…. N-acetamido (2.95 Å), and Glu^{347}….N-acetamido (2.95 Å)
		Hydrophobic interaction	Trp^{136}	Trp^{123}
	+3	Hydrogen bonding	Asp^{147}…H$_3$N-(2.90 Å)	Glu^{53}…. H-O- (2.95 Å), Arg^{94} ….N-acetamido (2.91 Å), Asp^{122}….N-acetamido (2.95 Å), Arg^{148}….O-acetamido (2.96 Å), and Arg^{312}…. H-O- (2.90 Å)
		Hydrophobic interaction	Trp^{136}	
	+4	Hydrogen bonding	Glu^{53}…H$_3$N- (3.04 Å), Glu^{53}…H-O- (3.07 Å), Asp^{122}….H-O- (2.61 Å), and Asp^{135}…H-O- (2.69 Å)	Asp^{135}…H-O- (2.96 Å), and Asp^{147}…H-O- (2.95 Å)
		Hydrophobic interaction	-	Trp^{136}
	+5	Hydrogen bonding	Glu^{53}…H-O- (2.52 Å), Asn^{127}….H-O- (3.01 Å), and Glu^{347}…. H-O- (3.07 Å)	-
		Hydrophobic interaction	-	Phe^{84}, Trp^{136}
	+6	Hydrogen bonding	-	Asn^{336}….H-O- (2.90 Å)
		Hydrophobic interaction	-	Phe^{84}, Trp^{331}
